# Self-Perceived Independent Living Skills and Self-Determination as a Method of Evaluating a Residential Program in Young Adults With Autism Spectrum Disorder

**DOI:** 10.7759/cureus.18133

**Published:** 2021-09-20

**Authors:** Rohan S Janwadkar, Stephanie Byun, Alicia Rootes, Nirmala Prakash

**Affiliations:** 1 Office of Diversity and Inclusion, Florida Atlantic University, Charles E. Schmidt College of Medicine, Boca Raton, USA; 2 Department of Integrated Medical Science, Florida Atlantic University, Charles E. Schmidt College of Medicine, Boca Raton, USA

**Keywords:** autism spectrum disorder (asd), employment, self-determination, independent living skills, adolescent and young adults

## Abstract

Prior research shows that employment programs for individuals with autism spectrum disorder (ASD) fail to address ASD as a heterogeneous disorder and focus on specific ASD traits associated with difficulty in obtaining and maintaining employment. This study provides descriptive evidence that self-perceptions of self-determination improve in young adults with ASD who participate in a residential program that promotes Wehmeyer and Schalock’s essential characteristics of self-determined behavior: behavioral autonomy, self-regulated behavior, acting in a psychologically empowered manner, and self-realization. Qualitative surveys were administered to 60 participants (17-28 years old) on perceptions of self-determination, confidence in independent living skills, and program effectiveness regarding case management and sustainable employment. One-sided t-tests using pre- and post-program responses were assessed. Post- versus pre-program means were significantly higher in participants feeling confident to live alone (p = 0.0059). Findings suggest that programs adopting self-determined behavior may be more effective in increasing self-confidence for individuals with ASD. However, these findings warrant long-term analysis to assess the continuity of program success and sustained employment.

## Introduction

Autism spectrum disorder (ASD) is a lifelong neurological condition characterized by deficits in social communication and restricted, repetitive sensory-motor behaviors [1]. ASD characteristics present on a spectrum from very mild to severe [1] and become especially apparent when it comes to obtaining meaningful and sustainable employment [1,2]. Although individuals with ASD possess desirable employment skills such as attention to detail and strong work ethic [2,3], there is discordance with sustained employment. Data from the National Longitudinal Transition Study-2 (NLTS2) showed that young adults with ASD have the lowest rates of holding paid employment after high school when compared with young adults with other types of disabilities, such as learning disabilities and speech/language impairments [4]. Young adults with ASD are working less than full-time, have a fewer number and less variation of jobs, and receive significantly lower compensation compared to those with other disabilities [5]. Even individuals who present with milder ASD, who are of higher or at the same level of intellectual ability as persons without disability, report difficulty in finding appropriate employment and maintaining work [6,7]. Employment provides economic independence and reduces the financial burden on caregivers. With the increasing prevalence of children with ASD in the United States [8] and the greatest risk for disengagement in postsecondary activities (employment and/or education) two years after high school [9], there is an increased need for sustainable postsecondary career development programs for individuals with ASD.

There are many employment programs and interventions currently available for individuals with ASD. These programs focus on employment preparation to teach necessary social, communication, and vocational skills and job maintenance through behavior and task management. The interventions obtain employment preparation and job maintenance by utilizing video modeling, role-playing, audio coaching, behavior skills training, job site, and simulation training. However, systematic analyses on the efficacy and success of these programs [2,10] show that many programs focus on addressing specific ASD traits associated with difficulty in obtaining and maintaining employment [2]. In addition, these programs fail to address ASD as a heterogenic disorder and lack information on the general characteristics of participants [10]. The authors highlighted the need for programs that are comprehensive in addressing the dynamic nature of ASD and referenced the review of Shattuck et al. (2012) [11] to focus on exploring self-determination in employment promotion and sustainability [12].

Self-determined behavior is defined as the combination of skills, knowledge, and beliefs, which empowers an individual to participate in goal-directed, self-regulated, autonomous behavior [13]. Wehmeyer and Schalock (2001) defined four essential characteristics of self-determined behavior in individuals with disabilities: (1) behavioral autonomy, (2) self-regulated behavior, (3) acting in a psychologically empowered manner, and (4) self-realization. The promotion of self-determined behavior and its characteristics is linked to higher levels of education and employment [14,15] and should be incorporated in designing new employment programs for individuals with ASD. There is evidence that programs incorporating principles of self-determination, such as Project SEARCH, are effective transition-to-work programs for young adults with ASD [16]. Furthermore, addressing vocational, social, communication, and learning needs of individuals with ASD in addition to the Project SEARCH curriculum is more successful in attaining competitive integrated employment at or above minimum wage within one year of completing the program [17].

The primary aim of this study is to provide descriptive evidence that self-perceptions of self-determination in young adults with ASD are malleable in a short period of time through participation in an urban residential summer camp program. Secondary aims include identifying demographic characteristics of study participants and how the addition of individual case managers affects young adults with ASD in developing meaningful employment.

## Materials and methods

The present study was conducted as a pretest-posttest research design using surveys to evaluate a summer camp program for young adults with ASD. The residential program was created by a non-profit organization in 2015 to address the lack of employment opportunities for young adults on the autism spectrum leaving school and entering adulthood [18]. The residential program was developed to aid young adults with ASD and their families in accessing adult services, obtaining competitive employment, and fostering independent living habits through the use of an established curriculum with support from qualified educators, autism specialists, case managers, counselors, and peer mentors. The program is a 12-day residential experience in which young adults with ASD are hosted on a large urban undergraduate campus. The summer program was established and substantiated by the Council for Exceptional Children, National Council for Accreditation of Teacher Education (NCATE) Recommendations for Technology in Teacher Education, American Disability Association, United States Department of Labor Office of Disability Employment Policy, and the National Technical Assistance Center on Transition.

Participants

Participants were referred by a state Division of Vocational Rehabilitation office, and those interested in participating in the summer program submitted an online application. Minimum eligibility criteria for the program included (1) a documented history of ASD, intellectual or developmental disability by a state Division Vocational Rehabilitation office; (2) minimum age of 17 years; (3) ability to meet personal needs without assistance (dressing, showering, shaving, and toileting); (4) ability to self-administer any necessary medication; (5) no disruptive behavior difficulties or violent tendencies; (6) no history of running or leaving school/home unsupervised; (7) ability to participate in multiple 50-90-minute classes daily; (8) ability to navigate campus independently with guidance from counselors (involving a considerable amount of walking); (9) ability to live in a group dorm setting and compromise with roommates; (10) interested in learning steps to employment, social skills, postsecondary education opportunities, and independent living; and (11) participation in all activities and classes. Applications were reviewed by summer program staff, and 64 participants were identified and accepted in the study. Participants and family members attended an in-person orientation where all details of the summer program and the informed consent were explained. All participants (or legal guardians) were required to give written informed consent. The study was approved by the residential university’s Institutional Review Board (1409572-3). Of the 64 participants, two were excluded due to attrition, and two were excluded due to missing post-program survey completion. The final analytical dataset consisted of 60 participants.

Procedures

The intervention was divided into three 12-day sessions with roughly 20 participants, two case managers, and program staff in each session. Participants resided at a large undergraduate institution during the entire program duration under the supervision of program staff. Each participant was matched with a case manager. Case managers were qualified social workers with graduate degrees in special education and previous experience in clinical or rehabilitation settings. Program staff members were individuals who were required to complete a 20-hour online certification course that prepared them for interaction with young adults with ASD. The participants were split evenly such that each of the two case managers had a caseload of no more than 12 individuals for each session. The case managers were expected to have direct contact with the participants at least five times throughout the session and assessed the participants’ ability to live and function independently and their employability skills. National metrics, including the Holland Code and Career Index, were utilized in case manager assessments. Case managers were also expected to have monthly follow-ups with each of their assigned participants after the successful completion of the residential program.

Table 1 shows how the residential program addressed each of Wehymer and Shalock’s four essential characteristics of self-determined behavior.

**Table 1:** Essential characteristics of self-determination addressed by program components

Essential Characteristics	Description	Program Components
Behavioral autonomy	Choice making	Independence from familial support
Self-regulated behavior	Identifying goals that match talents, developing implementation plans	Career planning, problem-solving
Acting in a psychologically empowered manner	Self-advocacy, self-efficacy	Case managers
Self-realization	Knowledge of strengths and limitations	Reflection and adaptation

Behavioral autonomy was established by hosting participants in a large urban undergraduate institution without direct familial support. Participants were still under supervision by program staff members but were encouraged to embrace their independence from family members and were taught how to self-navigate daily life. Participants were also given options for different social activities to choose from on their own and incorporate into their schedules.

Self-regulated behavior was established by giving participants individual daily responsibilities. Responsibilities were related to personal hygiene, dressing and clothing care, and time management. Participants also engaged in problem-solving activities to learn stepwise approaches to problems to apply in scenarios after program completion.

Acting in a psychologically empowered manner was established through instruction in executive functioning from case managers, qualified special education instructors, and autism specialists. Lessons were taught by the way of modeling, simulation, and guided practice for participants to learn how to select and successfully monitor behaviors that facilitate the attainment of their chosen goals. Case managers specifically taught participants about specific laws and adult services that could benefit them in finding and maintaining meaningful employment.

Self-realization was established through reflection and team-building activities where participants were able to consider beneficial and non-beneficial aspects of their behavior and the consequences of their behavior. Participants also met with case managers to monitor their progress and reflect on areas of improvement.

Measures

Participants were administered program surveys that included questions focused on participant self-determination, confidence in independent living skills, and program effectiveness in terms of case management and meaningful employment. Survey questions were developed by the summer program staff with oversight by faculty and the Director of Evaluation and Assessment at an urban medical school. Survey questions included the following: (1) I feel confident enough to live alone, (2) I have learned the professional skills needed to get a job, (3) I feel comfortable meeting with/talking to my case manager, (4) I have gained one or more ideas to use in my everyday life, (5) I feel confident in succeeding at my job, and (6) I feel confident in achieving my career plan goals. Surveys were administered via Google Forms at baseline and upon completion of the summer program. Responses were recorded on a five-point Likert scale (1 = strongly disagree, 2 = disagree, 3 = neutral, 4 = agree, 5 = strongly agree).

Statistical analysis

A one-sided t-test using pre- and post-program survey responses was assessed. The authors chose to do a whole group analysis rather than a paired pre-/post-analysis because not all participants completed the program to provide post-program survey responses, which rendered the data unsuitable for paired analysis. Microsoft Excel version 16.37 (Microsoft Corporation, Redmond, Washington, USA) was used for all statistical analyses. A one-sided p-value < 0.05 was considered statistically significant.

## Results

Descriptive characteristics

The descriptive characteristics of the study participants are listed in Table 2. Of the 60 participants, 40 (67%) were males, 19 (32%) were females, and one participant preferred not to answer. The mean (SD) age was 20.23 (2.56) years, and 47% of participants were identified as non-white. There were 23 (38%) participants who reported first-time attendance in this summer camp program. A majority (70%) of the participants were high-school graduates upon program entrance. Half of the participants indicated “student” as employment status, and 13% of participants reported they were currently seeking work.

**Table 2:** Participant descriptive characteristics (N = 60)

Descriptive Characteristics	Participants
	n (%)
Gender	
Male	40 (67)
Female	19 (32)
Preferred not to answer	1 (1)
Age (y)	
Mean	20.23
Range	17-28
SD	2.563
Previous Attendance	
First time (no previous attendance)	23 (38)
1 previous attendance	22 (37)
2 previous attendances	15 (25)
Race/Ethnicity	
Caucasian	32 (53)
African American	8 (13)
Hispanic/Latinx	7 (11)
Native American	3 (5)
Asian	0 (0)
Other	10 (17)
Highest Education	
Some/No diploma	7 (11)
High-school graduate	42 (70)
Some college credit	5 (8)
Associate’s degree	2 (3)
Trade school graduate	2 (3)
Bachelor’s degree	2 (3)
No schooling	0 (0)
Employment	
Student	30 (50)
Employed for wages	10 (17)
Out of work/Seeking	8 (13)
Out of work/Not seeking	6 (10)
Self-employed	3 (5)
Unable to work	3 (5)

Program survey responses

Results of the one-sided, two-tailed t-test using pre- and post-program survey responses are listed in Table 3. Overall, post-program means were higher than pre-program means, indicating a more positive response to self-determined behavior. The survey question, “I feel confident enough to live alone,” showed a significant response (p = 0.0059). Survey results also demonstrated a higher post-program mean (4.031 post-program mean versus 3.724 pre-program mean) for participants feeling more comfortable meeting and talking with their case managers, although the difference did not reach a significance (p = 0.065).

**Table 3:** Program survey responses

Survey Questions	Mean (Pre-program)	Mean (Post-program)	p-value
I feel confident enough to live alone.	3.034	3.578	0.0059
I have learned the professional skills needed to get a job.	3.621	3.891	0.091
Instruction sessions help me feel prepared to get a job.	3.586	3.844	0.093
I feel comfortable meeting with/talking to my case manager.	3.724	4.031	0.065
I have gained one or more ideas to use in my everyday life.	3.741	3.906	0.286
I feel confident in succeeding at my job.	3.759	3.906	0.393
I feel confident in achieving my career plan goals.	3.741	3.844	0.544

## Discussion

This study evaluated the immediate effects of a residential summer camp program in a large urban undergraduate institution. The program promoted the essential characteristics of self-determined behavior through independent living and employability skills in young adults with ASD in hopes of developing sustainable careers. The major finding in this diverse cohort of young adults with ASD was that participation in the residential summer camp program was associated with increased confidence for independent living immediately after program completion. Participants also felt greater comfort meeting and talking with case managers, although mean differences did not show significance.

The residential summer camp program used in this study identified and incorporated missing aspects from other vocational programs for young adults with ASD and integrated qualities necessary for adequate employment training, such as those seen in Project SEARCH with ASD supports [17]. Systematic reviews on vocational programs for individuals with ASD highlighted a need to address ASD as a heterogeneous disorder [2] to provide more information on the general characteristics of participants [10] and to incorporate both group-based and individual-based learning in a residential setting [18]. The program that was evaluated in this study utilized Wehymer and Shalock’s four essential characteristics of self-determined behavior to address ASD as a heterogeneous condition and focus interventions on lifelong independent living skills in addition to employment training. The program was also unique as participants lived on-site and were given 12 days of continuous education in both individual and group settings. The addition of a case manager provided individualized interaction and allowed participants to build longitudinal mentorship while engaging in meaningful conversations. Case managers had direct contact with the participants at least five times throughout the program and assessed the participants’ ability to live and function independently and their employability skills. Case managers were then able to identify individual interests, strengths, and weaknesses to find optimal and meaningful employment.

While many of the current vocational programs lacked descriptive characteristics of the participants [10], this study was able to identify basic demographic information to assess the current status and needs of young adults with ASD. Around one-third of the participants were identified as females, which is higher than most studies with predominantly male participants [18]. Females with ASD are underdiagnosed [19], and there is limited information on the specific needs of young adult females with ASD as it pertains to independent living and obtaining employment. Most of the participants (70%) were high-school graduates with only 22% of them employed for wages/self-employed. There is increasing evidence showing reduced to no day-time activities and services beyond high school in association with an increased risk for behavioral and mental health difficulties in young adults with ASD [14,15]. Studies on the National Longitudinal Transition Study-2 (NLTS2) dataset also show the first two years after high school as the most vulnerable time for young adults with ASD to not be involved in employment or higher education activities [9]. These statistics highlight the importance and need for vocational programs during this time of transition into adult life and the workforce.

Limitations and future research

There are limitations to this study that should be considered. First, the small sample size limited the analysis to a one-sided, two-tailed t-test. While this statistical test was chosen for attrition reasons, survey results were limited to whole group intra-mean differences. There was also no control group to establish inter-mean differences and to assess whether an increase in independent living skills would have occurred regardless of attending the summer camp program. The extensive eligibility criteria of this program limited participants to those at a higher functioning level on the ASD. Although the strict admission criteria limited generalizability to all individuals with ASD, there is still a need for vocational programs for higher functioning ASD adults [6]. Lastly, the results of this study were limited to immediate subjective effects after program completion. The survey only assessed subjective, self-reported feelings of whether participants with ASD felt more confident or comfortable in finding and maintaining employment. Therefore, the long-term effects of the study and whether the summer camp program is effective in finding long-term sustainable employment warrant further research.

Study results suggest that residential vocational programs for young adults with ASD who incorporate characteristics of self-determination provide short-term benefits in independent living skills. Future studies, including further follow-up and evaluation of this study cohort, are needed to obtain objective data on job placement and sustained employment rates. Additional qualitative metrics, such as interviews and focus groups, can further explain study findings and identify which aspects of the summer camp program correspond to better employment outcomes. The additional qualitative metrics can also help to elucidate specific needs of female young adults with ASD as it pertains to independent living, self-determination, and employment. Overall, future studies could utilize larger sample sizes to investigate the effects of similar residential programs across broader, more diverse populations of young adults with ASD. 

## Conclusions

This residential summer program provides a unique opportunity for young adults with ASD to become self-sufficient and live independently, with the goal of these skills translating to better employment opportunities and increased job performance. The program offers a simulation for participants to practice employment skills before entering the workforce where consequences for mistakes are often much higher. The results of the study suggest that residential programs incorporating characteristics of self-determination offer young adults with ASD the skills to live independently. Future studies should expand on the long-term effects of residential programs and incorporate greater sample sizes. Increased representation of a broader spectrum of ASD persons based on race, gender, and ethnicity is also needed.
